# Abortion and Contraception as Medical and Social Problems of Modern

**Published:** 2018-06

**Authors:** Assiya TURGAMBAYEVA, Zaituna KHAMIDULLINA, Assel BAUBEKOVA, Yelena DUDNIK, Marina ZHANALIYEVA, Lazat KASAEVA, Zhanyl SEIDAKHMETOVA, Nazira KAMZAEVA

**Affiliations:** 1. Dept. of Public Health N1, Medical University JSC, Astana, Kazakhstan; 2. Dept. of Obstetrics & Gynecology, Medical University JSC, Astana, Kazakhstan; 3. Dept. of Normal Anatomy, Medical University JSC, Astana, Kazakhstan; 4. Dept. of Therapeutic Disciplines, South Kazakhstan State Pharmaceutical Academy, Shimkent, Kazakhstan; 5. Corporate Fund “University Medical Center” National Research Center of Motherhood and Childhood, Astana, Kazakhstan

## Dear Editor-in-Chief

The outbreak in the twentieth-century sexual revolution gave rise to the acutest contradictions in the views and thus the controversy and conflict of opinion. First, perhaps, they touched the attitude towards abortion. In the Christian world against abortion categorically Catholic Church has acted yet in 1987. The Vatican issued a special instruction on this issue, and at the Cairo Conference in 1994 again spoke as sharply (1,2). Against abortion and family planning in general and serves the majority of the Muslim countries. The Protestant and Orthodox countries treat them far more tolerant. In total, annually takes place approximately 60 million in the world.

On the incidence of abortion can be judged as a whole on the state of health of the population, and the relation of the state to maternal and child issues.

Unfortunately, in many countries for many years abortion remains the primary method of birth control, and their number in the world is about 40–50 million. Annually, half of these abortions performed under unsafe conditions. Abortion annually leads to 70 thousands of maternal deaths and 5 million. Women get temporary or permanent disability.

Table 1 gives an idea of the relationship between fertility and abortion rates in some countries. The correlation coefficient (ρ), equal to 0.6, demonstrated the direct relationship between the average power of fertility rate and abortion rate.

**Table 1:** The frequency and the frequency of abortion infertility in some countries

***Country***	***Index of fertility (2014)***	***The frequency of abortions (per woman)***
Bulgaria	1.53	23
Canada	1.59	13
Germany	1.47	7
Denmark	1.69	14
Finland	1.39	11
France	2.01	17
Italy	1.37	10
Hungary	1.50	21
Japan	1.40	13
Netherlands	1.78	9
Norway	1.86	13
Switzerland	1.54	7
USA	2.01	20

The first contraceptive pill was created in 1954, Gregory Pincus in the United States. Contraceptive Efficacy was evaluated by the index of Pearl («failure rate»). The lower the score was the more reliable method of contraception. For example, if out of 100 women who used throughout the year by the same method of contraception, pregnant three, Pearl index for this method is equal to three. The lowest Pearl Index - using combined oral contraceptives and intrauterine hormonal spiral −0.1, and 150 million women in the world prefer to hormonal contraception (2.3).

“More than 99% of women aged 15–44 yr who had ever-sexual contact with a man who used at least one contraceptive” (5).

In the first place on the frequency of use of oral contraceptives were − 11.7 million women, sterilization −10.3. More educated women are more likely to use birth control pills, while the less educated more likely to resort to sterilization.

To date, well-researched abortion causes childbirth preferences, among which the first place is expressed in the majority of modern families installed on reduced lifetime fertility. The second and third place among the reasons for choosing abortion occupy unfavorable family relationships and the impossibility of combining his studies with the birth and upbringing of the child and in young families that reason when aborting a first pregnancy main.

With increasing age increases the probability of having a history of induced abortion women: if under the age of 20 yr only 15% of women who had an induced abortion, then to 25 yr already 30%, to 30 yr 80% of women (6). By the end of the reproductive period for every woman have an average of 3.6 abortions.

In the course of a large study conducted in the state of Georgia, USA has formed three groups: using modern contraceptives, using traditional contraceptives and not to use contraceptives. The use of contraceptives has increased over the years of observation by 23% (1999–2005.); at the same time, the frequency of abortions in couples over the years has decreased by 15% from 203 to 172 per 1000 woman-years (3).

Abortion is a very topical issue in Kazakhstan, despite the tendency to reduce them. Lack of awareness about the methods of contraception and the limited variety of reasons, early sexual activity increases the risk of pregnancy and lead to abortion in adolescent girls.

**Fig. 1:**
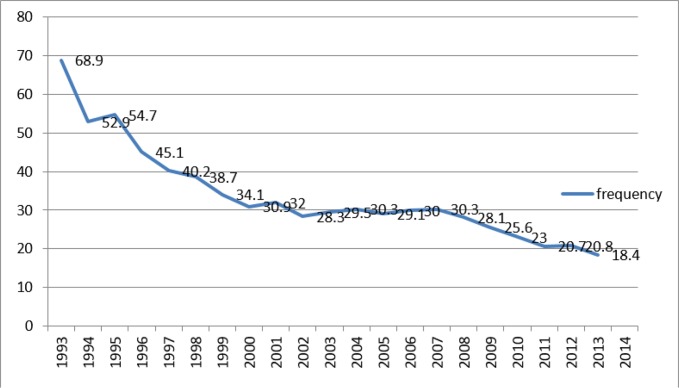
The dynamics of the frequency of abortion in the Republic of Kazakhstan for the period from 1993–2014 (Per 1000 women of childbearing age)

In Kazakhstan, there is a clear downward trend in the indicator (4) the number of abortions per 1000 women of childbearing age from 68.9 in 1993 to 18.4 in 2014 and to increase the number of women using modern contraceptives (Fig. 1).

Thus, the data of a sociological survey conducted in Kazakhstan showed that in 27.7% of cases, the conversation with the doctor persuaded the young women in the appropriateness of the pregnancy and the rejection of abortion. This Figure, despite the relatively small amount, can be evaluated positively, because such a conversation helped prevent abortion every sixth young woman.

## References

[1] Serbanescu F Stupp P Westoff C (2010). Contraception matters two approaches to analyzing evidence of the abortion decline in Georgia. Int Perspect Sex Reprod Health, 36(2):99–110. 20663746 10.1363/ipsrh.36.099.10

[2] Mosher WD Jones J (2010). Use of contraception in the United States: 1982–2008. Vital Health Stat 23,(29):1–44. 20939159

[3] Matthews TJ Hamilton BE (2009). Delayed childbearing: more women are having their first child later in life. NCHS Data Brief, (21):1–8. 19674536

[4] Kulov D.B. Problems of women’s reproductive health in Kazakhstan // Problems of reproduction. Almaty - 2010 - № 3 - P. 24–27.

[5] Daniels K Mosher WD Jones J Contraceptive methods women have ever used: United States, 1982–2010, National Health Statistics Reports, 2013, No. 62,http://www.cdc.gov/nchs/data/nhsr/nhsr062.pdf. 24988816

[6] Manuilova IL Sotnicova EI Troyickaya IA Krutcovscaya NP // Bulletin of obstetrics and gynecology. - 1990 - № 8 - P. 37–40. https://medi.ru.

